# Prevalence and Antibiogram Profiling of *Escherichia coli* Pathotypes Isolated from the Kat River and the Fort Beaufort Abstraction Water

**DOI:** 10.3390/ijerph110808213

**Published:** 2014-08-12

**Authors:** Nolonwabo Nontongana, Timothy Sibanda, Elvis Ngwenya, Anthony I. Okoh

**Affiliations:** Applied and Environmental Microbiology Research Group, University of Fort Hare, Private Bag X1314, Alice 5700, South Africa; E-Mails: TSibanda@ufh.ac.za (T.S.); ENgwenya@ufh.ac.za (E.N.); AOkoh@ufh.ac.za (A.I.O.)

**Keywords:** Kat River, *E. coli* pathotypes, antibiogram, public health, surface water

## Abstract

*Escherichia coli* is a widespread bacterium encompassing a variety of strains, ranging from highly pathogenic strains, causing worldwide outbreaks of severe diseases to avirulent, well characterized safe laboratory strains. This study evaluated the prevalence and antibiogram profiles of *E. coli* pathotypes isolated from the Kat River and Fort Beaufort abstraction water. A total of 171 out of 278 confirmed *E. coli* isolates were positive for at least one pathogenic determinant and these included enteropathogenic *E. coli* (6%), enterotoxigenic *E. coli* (47%), uropathogenic *E. coli* (2%), neonatal meningitis *E. coli* (5%), diffusely adherent *E. coli* (1%) and enterohaemorrhagic *E. coli* (1%). Interestingly, enteroinvasive and enteroaggregative* E. coli* were not detected*.* The phenotypic antibiogram profiles of the isolates revealed that all were resistant to penicillin G, while 98% and 38% of the pathotypes were resistant to ampicillin and trimethoprim-sulphamethoxazole, respectively. About 8% of the isolates were resistant to streptomycin. More than half of the isolates exhibited multiple antibiotic resistance with 44% being resistant to three antibiotics and 8% resistant to four antibiotics. We conclude that the Kat River is a reservoir of potentially virulent antibiotic resistant *E. coli* strains that can cause serious health risks to humans who drink raw water from this river, or in the case that consumption of treated drinking water coincides with failed drinking water processes.

## 1. Introduction

The use of antimicrobial agents plays a critical role in reducing morbidity and mortality due to communicable diseases. However, the emergence and spread of resistance to many of these antimicrobial agents is reducing their effectiveness. Reports from different parts of Africa have observed temporal trends in the prevalence of antibiotic resistance among enteric organisms, such as *E. coli* and *Shigella* [1,2]*.* Studies during the last 15 years show increasing resistance to commonly used antimicrobials such as trimethoprim-sulphamethoxazole (TMP-SMX, also known as cotrimoxazole), ampicillin, tetracycline and chloramphenicol [1,2].

*Escherichia coli* infections usually result when water and/or food contaminated with the bacterium is consumed. The general symptom of the infection is mostly diarrhea, which can cause death in immuno-compromised individuals such as the very young and the elderly, due to dehydration from prolonged illness [3]. *E. coli* O157:H7 is one of the *E. coli* strains which is now well-recognized as a cause of serious, and sometimes fatal, human illness. Despite the fact that *E. coli* O157:H7 infections are linked to consumption of infected dairy products such as raw cow milk, different sources of infection have been implicated, including leafy vegetables and water [4]. In the United States, the first reported drinking water outbreak of *E. coli* O157:H7 infections occurred in 1989 in rural Missouri [5]. Since this outbreak, many others have been associated with drinking water [6]. These outbreaks have led to the increased use of antibiotics to treat infections. The use of antibiotics in medicine and their applications in animal husbandry has brought about phenotypic changes, often due to chromosomal mutations, and antibiotic resistance in *E. coli* has been globally identified in isolates from environmental, animal and human sources [7]. Kinge* et al.* [3] report that *E. coli* has been linked to well-known antibiotic-resistant gene pools and that these genes are transferred into the normal flora of humans and animals, where they exert a strong selective pressure for the emergence and spread of resistance in *E. coli* strains. Inevitably, they discover their route into nature’s turf through wastewater, compost and sewage slime. In much of the developing world without access to good quality medicines, infections continue to be the major killers, and in all countries, infections with resistant microorganisms are a major cause of death [8].

Antimicrobial resistance is a public health threat and characteristic of pathogens causing different diseases. It is generally not a problem of disease pathology but one of limited therapy options [9], thus containment strategies must be adapted to the needs of specific disease control and treatment programs. Antibiotic-resistant bacteria have been found in a surprisingly diverse range of environments, including clinics, animal pens, orchards, aquaculture, food, sewage as well as chlorinated and unchlorinated water supplies [10]. Bacteria are a common contaminant worldwide; and the release of human and animal wastes into the environment exacerbates bacterial contamination, especially of aquatic milieu. Increased resistance to antibiotics may pose a challenge for the effective treatment of bacterial infections [11].

Clinicians use antibiograms to assess local susceptibility rates, as an aid in selecting empiric antibiotic therapy, and in monitoring resistance trends over time within an institution. An antibiogram shows the aggregate number of bacteria tested against antimicrobials and incorporates the extent of bacterial isolates vulnerable to every antimicrobial operator tested [12]. They lend information that can be used to raise awareness about resistance problems, support the use of optimal empiric treatment, and identify opportunities to reduce inappropriate antibiotic usage and to discover success of such efforts [12,13,14].

The most common methods utilized to measure the* in vitro* vulnerability of microorganisms to antimicrobial operators include the disk diffusion method, agar dilution, broth micro-dilution, and testing by antimicrobial gradient agar strips (E-test method). The disk diffusion (qualitative test) method can be used to estimate the* in vivo* adequacy of numerous antibacterial agents by measuring the zone of inhibition diameters and comparing it to the standards described by CLSI. In clinical microbiology laboratories, the antimicrobial gradient method is used routinely for testing common, rapidly growing, and certain fastidious bacterial pathogens [15]. The antimicrobial gradient diffusion method uses the principle of establishment of an antimicrobial concentration gradient in an agar medium as a means of determining susceptibility. The aim of this study was to assess the prevalence and antibiogram profiles of *E. coli* pathotypes isolated from the Kat River and Fort Beaufort abstraction water.

## 2. Experimental Section 

### 2.1. Description of Study Site

**Figure 1:**
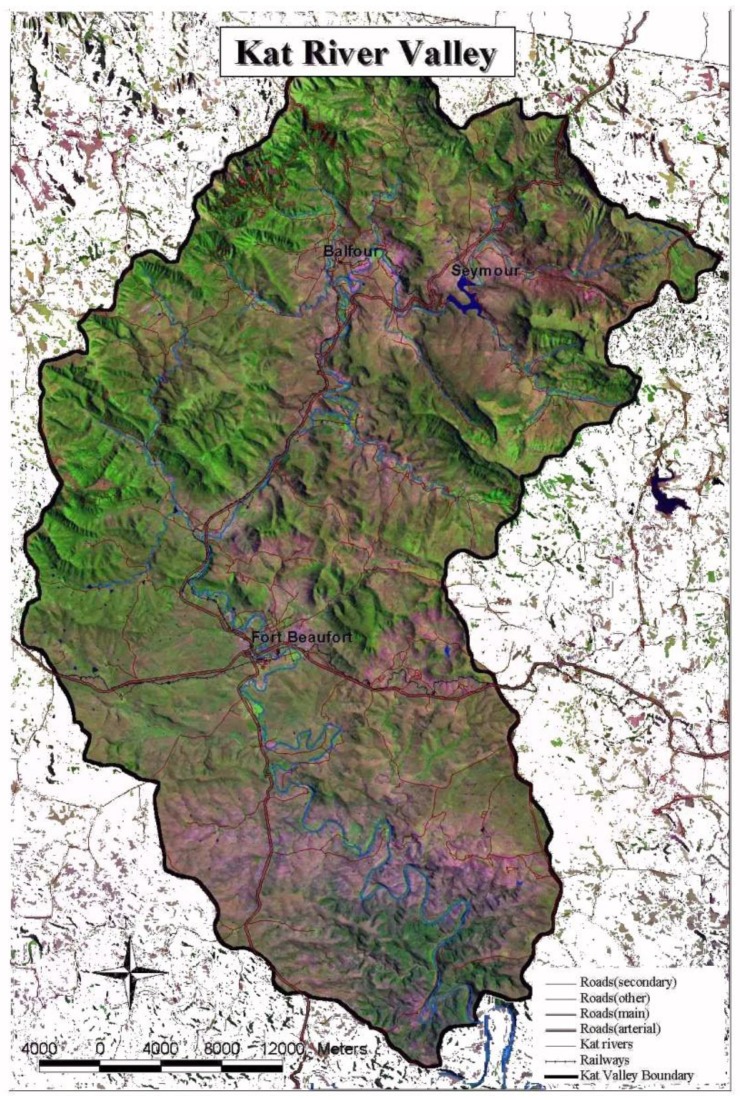
Kat River flowing through the Kat River valley.

Figure 1 below shows the Kat River catchment which covers an area of approximately 1700 km^2^. It is found in the Eastern Cape Province of South Africa, and has its source in the Katberg Mountains. The river then flows south for about 90 km before discharging directly into the Indian Ocean. Water from this river is mainly used for irrigation of large citrus orchards, as well as a source of abstraction water for the Fort Beaufort municipal waterworks. Fort Beaufort is a town situated in the Eastern Cape Province of South Africa that lies at geographical co-ordinates 32°47'0"S, 26°38'0"E. Currently, raw water is extracted from the Kat River at a barrage and transferred to a raw water storage dam situated near the water treatment works.

The Kat River Valley is characterized by a variety of land uses, ranging from export orientated citrus farming, commercially oriented rangeland stock farming to small-scale vegetable and crop production as well as stock farming [16]. Commercial farmers are mainly located in the Middle Kat and Lower Kat, whereas smallholders and emerging farmers mostly practice agriculture in the Upper catchment. The catchment is home to ≈178,000 people, only 10% of whom reside in Fort Beaufort, the only urban centre in the Kat River region. The rest of the population reside in rural, remote villages, where only a few have access to potable water, and on commercial farms, where they work as farm labourers [17]. 

### 2.2. Sample Collection

Samples were collected using sterile 1.75-litre plastic bottles once a month over a three month period (August 2012 to October 2012) from five sampling points selected along the course of the Kat River (Figure 1). All samples were collected at a depth of about 15 cm, in the direction of water flow, and transported in cooler boxes with ice to the Applied and Environmental Microbiology Research Group (AEMREG) laboratory at the University of Fort Hare for analysis within four hours of collection.

On arrival to the laboratory, water samples were serially diluted and 100 mL aliquots of each dilution were filtered through a 47 mm diameter, 0.45 µm pore sized membrane filters according to Standard Methods [18]. The membrane filters were then sterilely transferred onto *E. coli* chromogenic agar using sterile forceps, and incubated at 37 °C for 24 h. After incubation, only the blue colonies were enumerated as presumptive *E. coli*. These isolates were subjected to Gram’s staining, oxidase and catalase tests. Only isolates that were Gram negative, oxidase negative and catalase negative were preserved on glycerol stock at −80 °C for further analysis.

### 2.3. Pathotype Identification

The stored isolates were re-activated by culturing them in nutrient broth at 37 °C for 24 h before being transferred to nutrient agar plates to check for purity. Four to five lone colonies of each purified bacterial isolate were then suspended in 200 µL of sterile distilled water and centrifuged for 5 min at 13,400 rpm in a microcentrifuge. The supernatant was decanted and the cells re-suspended in 200 μL of sterile nuclease free water, vortexed briefly and boiled at 95 °C for 15 min followed by high speed centrifugation at 12,000 rpm for 10 min [19]. The supernatant which contained the DNA was transferred into new, DNase free tubes for use in polymerase chain reaction (PCR) assays or kept at −20 °C until ready for use. PCR was done following the method of Moyo* et al.* [20], with some modifications as follows: In a single PCR reaction tube the following reagents were added for each isolate: 12.5 µL of master mix (containing dNTPs, DNA Taq polymerase, MgCl_2_ and PCR buffer), 5 µL of sample DNA, 0.5 µL of the forward and reverse primers (100 µM), and 6.5 µL of nuclease free water to make a 25 µL reaction volume. Target DNA was then amplified using the following protocol; denaturation at 94 °C for 3 min followed by 35 cycles of melting at 94 °C for 1 min, annealing at pathotype specific temperatures (Table 1) for 1 min and extension at 72 °C for 1 min, and a final extension step at 72 °C for 5 min. Target genes and primers sequences for the detection of the pathotypes as well as the expected amplicon sizes and annealing temperatures are as listed in Table 1.

**Table 1:** *E. coli* target genes and primer sequences used to identify/characterise *E. coli* pathotypes.

Target Strains	Target Genes	Primer sequence (5’→3’)	Amplicon Size (bp)	Annealing Temp (ºC)	References
EHEC	*Shiga toxin 1 (stx1)*	F-CAGTTAATGTGGTGGCGAAGG R-CACCAGACAATGTAACCGCTG	348	55	[21]
	*Shiga toxin 2 (stx2)*	F-ATCCTATTCCCGGGAGTTTAC G R-GCGTCATCGTATACACAGGAGC	584	55	[21]
	*flicH7*	F: GCGCTGTCGAGTTCTATCGAGC R: CAACGGTGACTTTATCGCCATTCC	625	55	[22]
EPEC	*Intimin (Eae) gene*	F-TCAATGCAGTTCCGTTATCAGTT R-GTAAAGTCCGTTACCCCAACCTG	482	54	[23]
ETEC	*Heat-labile toxin (lt)*	F-GCACACGGAGCTCCTCAGTC R-TCCTTCATCCTT TCA ATG GCT TT	218	58	[23]
EIEC	*Invasin plasmid antigen (ipaH)*	F-CTC GGC ACG TTT TAA TAG TCTGG R-GTGGAG AGC TGA AGT TTC TCTGC	933	53	[24]
EAEC	*Aggregative adherent fimbriae (aafII)*	F-CACAGGCAACTGAAATAAGTCTGG R-ATT CCC ATG ATG TCA AGC ACT TC	378	56	[24]
DAEC	*F1845 Fimbriae (daaE)*	F-GAACGT TGG TTA ATG TGG GGT AA R-TAT TCA CCG GTC GGT TAT CAG T	542	54	[24]
UPEC	*Pyelonephritis-associated pili (pap)*	F-AAC CTGGCTTACGCAACTGTACCC GT R-CTG CAA AAT CAT GGA T	585	58	[21]
NMEC	*Invasion of brain endothelial (IbeA) gene*	F-TGGAACCCGCTCGTAATATAC R-CTGCCTGTTCAAGCATTGCA	900	58	[21]

### 2.4. Antibiotic Susceptibility Test

Antimicrobial susceptibility testing was done on Mueller-Hinton agar (MHA) (Merck biolab, Gauteng) by the standard disc diffusion method recommended by the Clinical and Laboratory Standards Institute [25]. Fresh cultures (about 22 h old) were transferred into test tubes containing 5 mL sterile normal saline. The turbidity of the suspension was adjusted to 0.5 McFarland standards (equivalent to 1.5 × 10^8^ CFU/100 mL). Sterile swabs were soaked into the bacterial suspensions and used to inoculate the MH agar plates by spreading uniformly on the surface of the agar, after which five antibiotic discs were placed equidistant from each other on the agar surface and the plates were incubated at 35 ± 2 °C for 18 to 24 h. The antibiotics used in this study are shown in Table 2. A total of 278 isolates were used for antimicrobial susceptibility testing against the panel of 10 test antibiotics. After incubation, the plates were examined for zones of inhibition which were then measured and interpreted using the minimal inhibitory concentration (MIC) breakpoints for Enterobacteriaceae [25].

**Table 2:** Minimal Inhibitory Concentration (MIC) Breakpoints for Enterobacteriaceae. Source: (CLSI [25]).

Test/Reportgroup	Antimicrobialagent	DiskContent (µg)	Zone Diameter Breakpoints, Nearest Whole mm			MIC Interpretive Standard (µg/mL)		
			S	I	R	S	I	R
**A**	Ampicillin	10	≥17	14–16	≤13	≤6	16	≥32
**A**	Gentamycin	10	≥15	13–14	≤12	≤4	8	≥16
**B**	Amikacin	30	≥17	15–16	≤14	≤16	32	≥64
**O**	Streptomycin	10	≥15	12–14	≤11	-	-	-
**O**	Kanamycin	30	≥18	14–17	≤13	≤16	32	≥64
**C**	Tetracycline	30	≥15	12–14	≤11	≤4	8	≥16
**B**	Ciproflaxacin	5	≥21	16–20	≤15	≤1	2	≥4
**U**	Norflaxacin	10	≥17	13–16	≤12	≤4	8	≥16
**B**	Trimethoprime-sulfamethoxazole	1.25/23.75	≥16	11–15	≤10	≤2/38	-	≥4/76
**C**	Chloramfenicol	30	≥18	13–17	≤12	≤8	16	≥32

### 2.5. Evaluation of Antibiotic Resistance Genes

Following* in vitro* susceptibility testing, the relevant antibiotic resistance determinants were evaluated using PCR. The target antibiotics resistance genes are listed in Table 3.

**Table 3:** Details of target antibiotic resistance genes.

Antibacterial Agent	Resistance Gene	Sequence	Amplicon size (bp)	Annealing Temp. (°C)	References
Streptomycin	aadA1	(F)TATCCAGCTAAGCGCGAACT	147	58	[26]
		(R)ATTTGCCGACTACCTTGGTC			
β-Lactams	Bla	(F)TCGCCTGTGTATTATCTCCC	198	52	[26]
		(R)CGCAGATAAATCACCACAATG			
Tetracycline	tetA	(F)CCTCAGCTTCTCAACGCGTG	402	56	[27]
		(R)GCACCTTGCTGATGACTCTT			
TMP-SMX	dfrA1	(F)GGAGTGCCAAAGGTGAACAGC	721	45	[28]
		(R)GAGGCGAAGTCTTGGGTAAAAAC			

## 3. Results and Discussion

### 3.1. Enumeration of E. coli 

**Figure 2:**
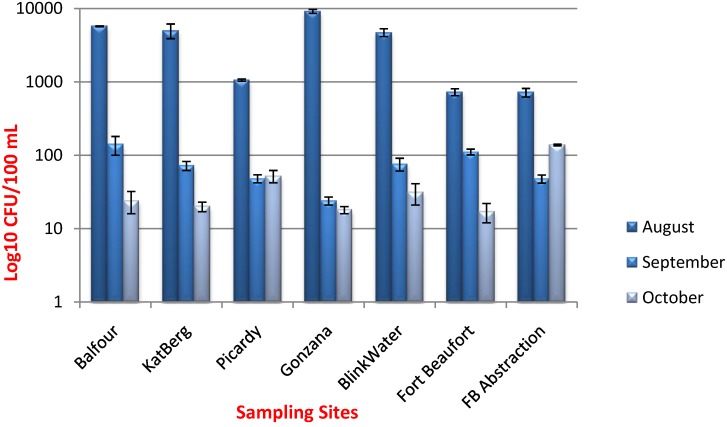
*E. coli* counts from all sampling sites for the month of August, September and October.

The *E. coli* counts for all the sampling sites ranged from 717 to 9100 CFU/100 mL; 24 to 140 CFU/100 mL and 17 to 137.6 CFU/100 mL for August, September and October, respectively. Taking into account that some poor rural people in the Kat River catchment still rely directly on Kat River water for daily consumption and household use, these results indicated that all our sampling sites were of poor microbiological quality as the *E. coli* concentrations were higher than the acceptable maximum limit of 0 CFU/100 mL of drinking water prescribed by the DWAF of South Africa. Figure 2 presents the total presumptive *E. coli* counts for each sampling site for the three months in which samples were collected. 

Throughout the sampling period for this study, fresh and dry human and animal excreta were spotted along the shores of the river. This could explain the high faecal coliform counts that we obtained. Cattle are the reservoirs of *E. coli* O157:H7 and the presence of cattle dung on the shores or in the water which people drink only increases the chances of people getting exposed to enteric infections. The abundance also of human excreta along the banks of the river and the elevated *E. coli* concentrations in the river water is cause of concern taking into account that the Eastern Cape Province has been identified as one of the worst off provinces in South Africa in relation to resources, socio-economic status and burden of disease. Its major indicators include; the lowest proportion of people with access to potable water supplies, electricity and sanitation out South Africa’s nine provinces, the highest level of poverty, the highest number of infant deaths, and the second highest number of child deaths under the age of five in the whole of South Africa [29]. As of 1996, 45.1% of households in the Eastern Cape still used water from streams, rivers, boreholes, springs and dams/pools. Although this proportion dropped to 22.2% in 2011 [30], it still represents significantly higher pockets of the Eastern Cape population if considered by districts. In OR Tambo District for example, 36% of the population had no access to piped water as of 1997, and 28% made use of a river or stream, 2% made use of a spring, 2% of a borehole and 2% of a pool or dam where water is stagnant [31]. Pollution of surface water resources like Kat River and their subsequent contamination with potentially virulent enteric microorganisms poses a public health risk to the resident population either through ingestion or contact. 

### 3.2. Pathotype Characterization

**Figure 3:**
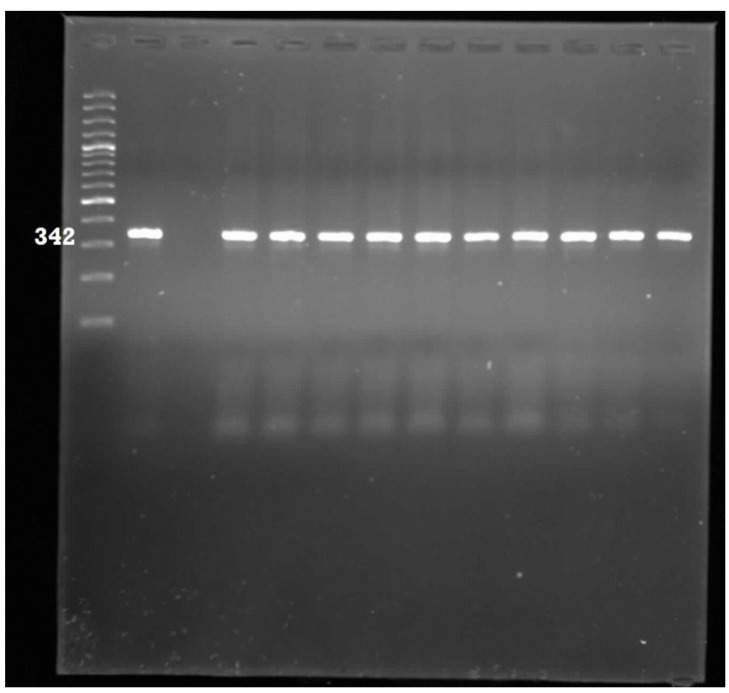
Gel electrophoresis for the detection of Neonatal meningitis *E. coli* amplicons. Lane 1: 100 bp ladder; Lane 2: Positive control; Lane 3: Negative control; Lane 4–13: Positive samples.

**Figure 4:**
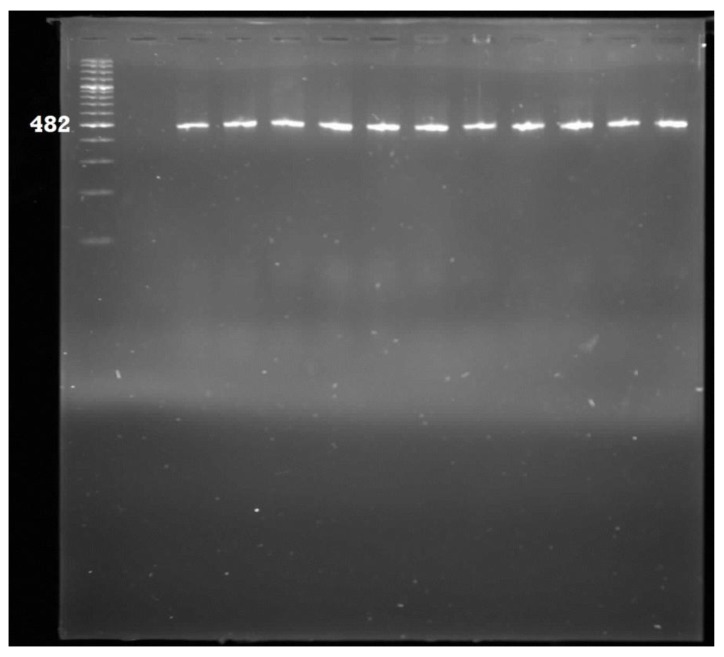
Gel electrophoresis for the detection of Enteropathogenic *E. coli* amplicons. Lane 1: 100 bp ladder; Lane 2: Negative control; Lane 3: Positive control; Lane 4–13: Positive samples.

**Figure 5:**
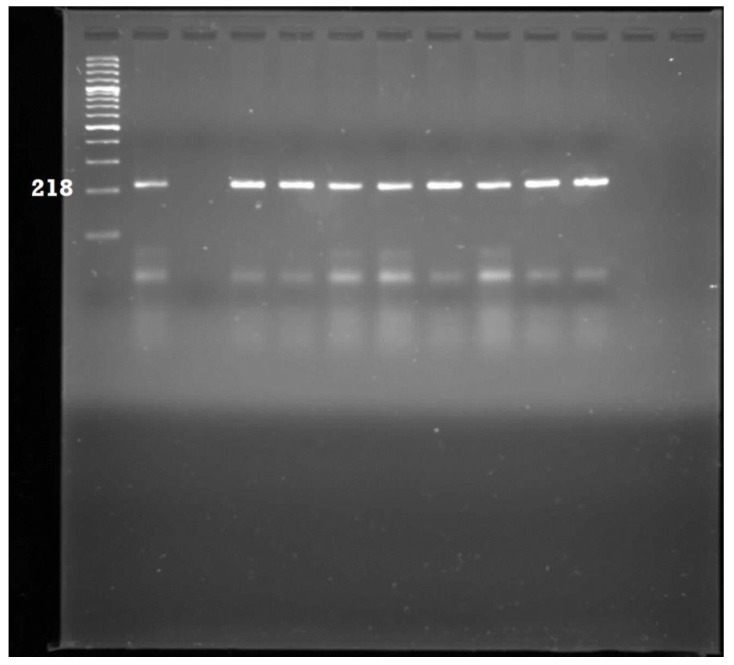
Gel electrophoresis for the detection of Enterotoxigenic *E. coli* amplicons. Lane 1: 100 bp ladder; Lane 2: Positive control; Lane 3: Negative control; Lane 4–11: Positive samples.

**Figure 6:**
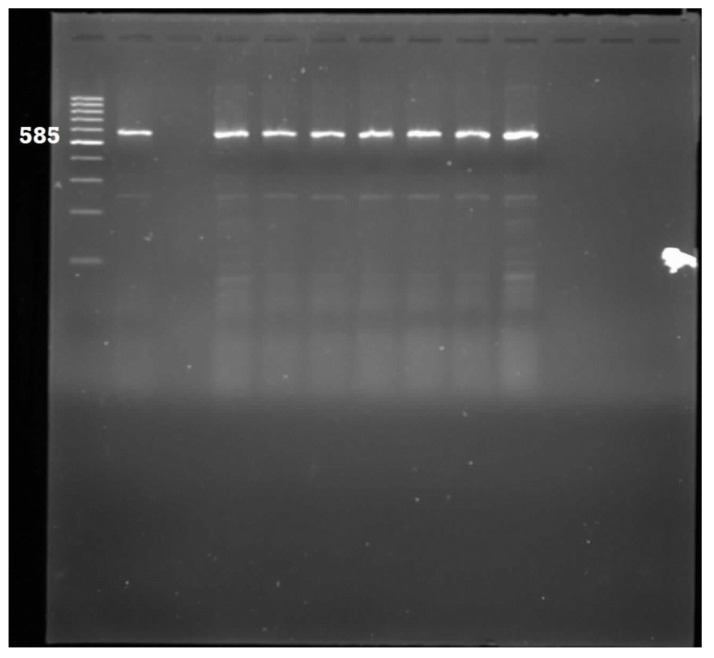
Gel electrophoresis for the detection of Uropathogenic *E. coli* amplicons. Lane 1: 100 bp ladder; Lane 2: Positive control; Lane 3: Negative control; Lane 4–10: Positive samples.

A total of 278 PCR-confirmed *E. coli* isolates were analysed for the presence of eight pathogenic genes (Table 1). The most prevalent pathotype detected was Enterotoxigenic *E. coli* which accounted for 47% of the isolates. This was followed by Enteropathogenic *E. coli* (6%); Neonatal meningitis *E. coli* (5%) and Uropathogenic *E. coli* (2%). The *flicH7* gene for Enterohaemorhaegic *E. coli* was detected in one isolate, same also for the diffusely adherent *E. coli* pathotype*.* The *aafII* (Enteroaggregative *E. coli*) and *ipa* (Entero-invasive *E. coli*) target genes were not detected in all the isolates. Some of the gel pictures for the PCR products of pathotype delineation are shown in Figure 3, Figure 4, Figure 5 and Figure 6. 

Overall, 70% of the presumptive isolates were confirmed to be *E. coli*. While this study did not include microbial source tracking (MST) to ascertain the specific source of the *E. coli* isolates recovered from the surface waters, it is highly likely that the isolates were from human and animal excreta. This assumption is supported by the fact that during our sampling regimes human and animal excreta were observed at the banks of the river and also that livestock were observed drinking at these water sources. This further implicates both humans and animals as potential sources for the recovered *E. coli* pathotypes.

### 3.3. Antibiotic Susceptibility Testing

The antibiotic susceptibility profile of the confirmed isolates is presented on Table 4. About 98% of the isolates were 100% susceptible to norofloxacin, while susceptibility to the other antibiotics were in the following order: amikacin (97%), ciprofloxacin (93%), streptomycin (77%), tetracycline (75%) and chloramphenicol (73%). All the isolates were 100% resistant to penicillin G, while 98% of the isolates were resistant to ampicillin. A number of the isolates exhibited resistance to streptomycin, tetracycline, trimethoprime-sulphametoxazole and the β-lactam class of antimicrobials. The resistance to these specific antimicrobials is sometimes encoded by plasmids, which may distribute resistance in susceptible bacteria through horizontal gene transfer [32,33,34]. Our findings also indicate that the *E. coli* recovered in this study expressed high levels of resistance to antimicrobials that are commonly used in clinical medicine. This could contribute to the spread and persistence of antimicrobial resistant bacteria and resistance determinants in humans and the environment.

**Table 4:** Antibiogram of *E. coli* isolated from Kat River and Fort Beaufort abstraction water.

Antibacterial Agent	Number of Isolates ( *n* = 278); Percentages in Parenthesis		
	Resistant	Intermediate	Susceptible
Ampicillin (AP)	272 (98%)	0 (0%)	6 (2%)
Penicillin G (PG)	278 (100%)	0 (0%)	0 (0%)
Tetracycline (T)	37 (13%)	33 (12%)	208 (75%)
Gentamycin (G)	0 (0%)	20 (7%)	258 (93%)
Chloramphenicol (C)	0 (0%)	75 (27%)	203 (73%)
Trimethoprim-Sulphamethoxazole (TS)	105 (38%)	23 (8%)	150 (54%)
Streptomycin (S)	22 (8%)	44 (16%)	212 (77%)
Ciprofloxacin (CIP)	0 (0%)	19 (7%)	259 (93%)
Norofloxacin (NOR)	0 (0%)	8 (2%)	272 (98%)
Amikacin (AM)	0 (0%)	9 (3%)	269 (97%)

One of the most important factors contributing to the spread of antimicrobial resistance in bacteria has been attributed to the fact that in most developing countries, diarrheal diseases are treated with an inadequate regimen of antimicrobials and often without first identifying the pathogen [35]. Use of antibiotics in animal husbandry as growth promoters could be another factor contributing to the recovery of resistant bacteria in these water sources as the gut microbial flora of these animals end up developing resistance to these antimicrobials, and passing the same to autochthonous bacteria in surface water systems. The dissemination of antimicrobial resistance in these pathogens may have potential negative clinical implications for therapeutic advancement. *E. coli* with multiple antimicrobial resistances in surface water and other environmental media have been reported [36,37]. The emergence of resistance and decreasing levels of susceptibility of *E. coli* isolates inhabiting aquatic niches to a wide spectrum of antimicrobials is a cause of concern as it may limit the availability of antimicrobials for clinical management of waterborne outbreaks in the future.

### 3.4 Antibiotic Resistance Determinants

The presence of antibiotic resistance genes encoding resistance to tetracycline, streptomycin and the β-lactam class of antibiotics was investigated and the results are presented in Table 5.

**Table 5:** Incidence of some antibiotic resistance determinants screened.

Antibiotic Determinant Screened	Resistance Encoded	Number of Isolates Tested	Positives (%)
*aadA*	Streptomycin	22	100
*tetA*	Tetracycline	37	0
*Bla*	β-Lactamase	272	54

All the isolates screened for the streptomycin resistance gene (*aadA)* were positive, while only 54% of the isolates tested positive for the β-lactam (*bla*) resistance gene. These findings show the distribution of antibiotic resistance genes which can possibly be carried on *E. coli* plasmids, emphasizing the potential to contribute to the efficient spread of antibiotic resistance. The prevalence of antibiotics or traces thereof in characteristic environments can challenge the populace flow and the physiology of natural microbial populations [36]. However, several reports indicate that the resistance genes currently present in human or animal associated microbiota are found in environments without antibiotic pollution [37,38,39]. This strongly supports the fact that resistant genes can persist and spread in the environment via horizontal gene transfer (HGT), thus, increasing the chances of pathogens acquiring resistance.

The dissemination of high levels of anti-microbials and resistance genes in characteristic biological communities is a late occasion in evolutionary terms. All things considered, both sorts of contamination can affect the structure and the action of ecological microbial populaces. Given that ecological microorganisms are the first wellspring of resistance genes procured through HGT by human pathogens [40] these changes are significant for the fate of human health.

## 4. Conclusions

We conclude that Kat River and Fort Beaufort abstraction waters are an important shared resource for people in the surrounding rural settlements from Seymour through to the Fort Beaufort communities. However, the presence of *E. coli* pathotypes and the multiple antibiotic resistance strains in these surface waters highlight the human health risk associated with exposure to the surface wasters. This suggests the need for adequate risk prevention strategies to protect the water and consequently public health which is a subject of intensive investigation in our group. 
